# Functional trajectories between 90 days and 1 year after intracerebral haemorrhage: a Swedish Stroke Register (Riksstroke) study

**DOI:** 10.1093/esj/aakag129

**Published:** 2026-10-28

**Authors:** Marcus Johnsson, Björn M Hansen, Trine Apostolaki-Hansson, Mats Pihlsgård, Bo Norrving, Teresa Ullberg

**Affiliations:** Department of Neurology, Skåne University Hospital, Lund, Sweden; Department of Clinical Sciences Lund, Lund University, Lund, Sweden; Department of Radiology, Skåne University Hospital, Lund, Sweden; Stroke Imaging Research Group, Department of Clinical Sciences Lund, Lund University, Lund, Sweden; Department of Neurology, Skåne University Hospital, Lund, Sweden; Department of Clinical Sciences Lund, Lund University, Lund, Sweden; Perinatal and Cardiovascular Epidemiology, Department of Clinical Sciences Malmö, Lund University, Malmö, Sweden; Department of Neurology, Skåne University Hospital, Lund, Sweden; Department of Clinical Sciences Lund, Lund University, Lund, Sweden; Department of Neurology, Skåne University Hospital, Lund, Sweden; Department of Clinical Sciences Lund, Lund University, Lund, Sweden

**Keywords:** functional outcome, ICH, longitudinal study, mRS, recovery trajectories, stroke registry

## Abstract

**Introduction:**

Functional outcome after ICH is commonly assessed at 90 days, although recovery and deterioration may continue beyond this conventional endpoint. Large-scale data describing within-patient functional trajectories beyond 90 days remain limited.

**Patients and methods:**

We conducted a register-based cohort study using the Swedish Stroke Register (Riksstroke), including adults with ICH between 2017 and 2021. Prestroke-independent 90-day survivors with available mRS data at both 90 days and 1 year were included. Functional trajectories were classified as improved, unchanged, worsened or died according to transitions across grouped mRS categories (0–2, 3, 4, 5 and 6 [death]). Multivariable logistic regression identified predictors of worsening or death and improvement.

**Results:**

Among 13,574 patients with ICH, 8583 prestroke-independent patients fulfilled eligibility criteria. Of these, 4797 were alive with available 90-day mRS data, and 3030 had complete mRS assessments at both 90 days and 1 year and were included in the primary analysis. Between 90 days and 1 year, 21.5% improved, 67.6% remained unchanged, 5.2% worsened and 5.8% died. Recovery at 1 year was most frequent among patients with mRS 3 at 90 days, of whom 62.4% achieved mRS 0–2, whereas 92.8% of patients with mRS 0–2 remained in the same category. Among patients with mRS 4–5 at 90 days, 20.4% improved to mRS 0–3 by 1 year. Increasing age and higher 90-day disability were associated with less favourable trajectories, whereas male sex was associated with greater likelihood of improvement.

**Conclusion:**

Among prestroke-independent 90-day survivors with complete functional follow-up, functional outcomes after ICH remained dynamic beyond 90 days, particularly among patients with moderate disability. Nearly two-thirds of patients with moderate disability at 90 days achieved mRS 0–2 by 1 year, whereas more than 9 in 10 patients who were already classified as mRS 0–2 remained so. These findings suggest that longer-term outcome assessment may provide additional clinically relevant information in future ICH studies and clinical trials.

## Graphical abstract

**Graphical abstract:**
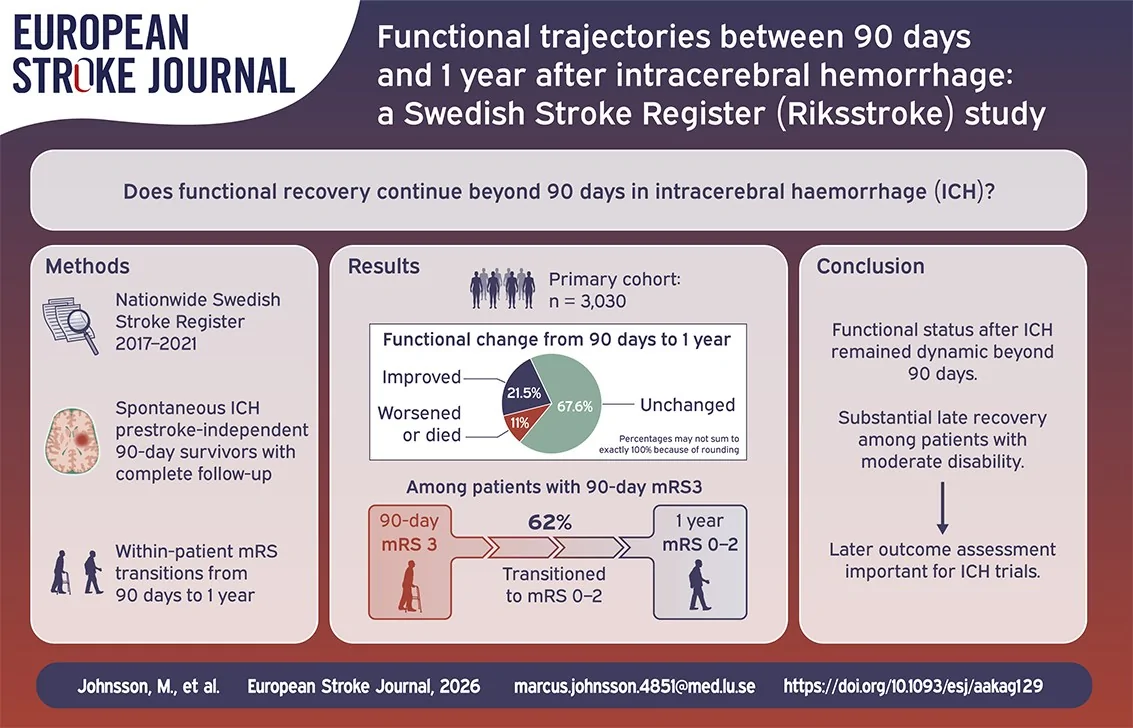

## Introduction

Intracerebral haemorrhage (ICH) is a severe form of stroke associated with high early mortality and substantial long-term disability among survivors.^1^^,^^2^ Functional outcome after stroke is most commonly assessed using the mRS at 90 days, a time point that has become the standard endpoint in both clinical trials and observational studies.^3^

However, currently available reports suggest that recovery after ICH may extend well beyond the acute and subacute phases, potentially reflecting a slower and more prolonged recovery trajectory than that of ischaemic stroke.^4^^,^^5^ In recognition of this, several contemporary randomised clinical trials in ICH have adopted 180-day or 1-year functional outcome assessments to better capture clinically meaningful recovery over time.^6–9^ Emerging evidence likewise suggests that functional recovery after ICH continues throughout the first year after stroke, with some patients demonstrating continued improvement beyond the conventional 90-day endpoint.^4^^,^^5^^,^^10^^,^^11^ Previous population-based and registry studies have also described long-term functional outcomes after ICH up to 1 year.^12–14^ However, dynamic within-patient transitions between disability states from 90 days to 1 year remain incompletely understood, as prior studies have often included selected populations, such as supratentorial ICH,^4^ focused primarily on favourable recovery or severe disability,^5^ or relied on dichotomised outcomes rather than transitions across the ordinal mRS.^11^ Consequently, longitudinal data from large ICH cohorts reflecting routine clinical practice remain limited.

In this cohort study, we aimed to characterise within-patient functional transitions across the ordinal mRS between 90 days and 1 year after ICH and to identify factors associated with subsequent improvement and worsening. By focusing on individual trajectories over time in a large ICH cohort, this study sought to provide a more detailed characterisation of functional recovery beyond the conventional 90-day endpoint and the increasingly adopted 180-day time point.

## Patients and methods

### Study design and data sources

This nationwide, register-based cohort study examined functional outcomes after ICH using prospectively collected data from the Swedish Stroke Register.^15^^,^^16^ Riksstroke is a national quality register including all 72 hospitals in Sweden admitting patients with acute stroke and captures the vast majority of hospitalised stroke cases nationwide. The study population comprised patients with ICH registered between 1 January 2017 and 31 December 2021. Functional outcomes were assessed using standardised follow-up questionnaires administered at 3 months and 1 year after stroke.

### Participants and follow-up

Eligible participants were adults (≥18 years) with a primary diagnosis of spontaneous ICH (ICD-10 code I61) recorded in Riksstroke between 2017 and 2021. To avoid duplicate inclusion, only the first recorded stroke event per individual during the study period was retained. The primary analyses were restricted to prestroke-independent patients (prestroke mRS ≤ 2) to facilitate interpretation of recovery related to the index ICH event rather than pre-existing disability and comorbidity. Individuals who died within 90 days were excluded from longitudinal analyses. The primary estimand was change in functional status between 90 days and 1 year among prestroke-independent 90-day survivors with evaluable mRS at both follow-up assessments.

### Variables

Baseline variables included age, sex, living situation, vascular risk factors, prestroke medication, clinical characteristics at admission, imaging findings and acute management. Vascular risk factors included prior stroke, previous transient ischaemic attack or amaurosis fugax, atrial fibrillation, hypertension, current smoking and diabetes mellitus. Prestroke medication included statins, antiplatelet therapy and oral anticoagulants, categorised as vitamin K antagonists or direct oral anticoagulants. Clinical variables included level of consciousness at admission, stroke severity assessed using the NIHSS, admission to a stroke unit and length of hospital stay. Imaging variables included ICH location, categorised as deep, lobar, supratentorial unspecified, brainstem, cerebellar, multiple or other; and presence of intraventricular haemorrhage. Acute management variables included neurosurgical treatment and reversal of anticoagulation therapies among patients receiving oral anticoagulants before stroke. During the study period, neurosurgical management primarily comprised conventional haematoma evacuation, ventricular drainage and intracranial pressure monitoring; minimally invasive haematoma evacuation was not routinely used during the study period. Riksstroke records whether neurosurgical treatment was performed but does not distinguish between specific procedures. Functional outcome was assessed at 90 days and 1 year and categorised as mRS 0–2, 3, 4, 5 and 6 (death). The 90-day mRS was reconstructed using a previously validated translation algorithm based on activities of daily living items available in Riksstroke, including mobility, toileting, dressing, living conditions and dependency on next of kin.^17^ The underlying functional items were collected locally through standardised Riksstroke follow-up and subsequently used to reconstruct mRS categories centrally using the validated algorithm. The follow-up questionnaires could be completed by the patient alone, with assistance, or by a proxy. Patients who were independently mobile and independent in toileting and dressing, without dependency in living conditions or support from others, were classified as mRS 0–2. Patients with some dependency in mobility, living conditions or support from others, while remaining independent in toileting and dressing, were classified as mRS 3. Requirement for assistance with mobility, toileting or dressing corresponded to mRS 4, whereas combinations indicating severe dependency across these domains corresponded to mRS 5. Vital status at both follow-up time points was ascertained independently of questionnaire response using national mortality data, and patients who had died were classified as mRS 6.^18^ Prestroke and 1-year mRS categories were reconstructed using the same item-based approach to ensure consistency across time points. Because the reconstruction algorithm does not reliably distinguish between mRS 0, 1 and 2, these categories were analysed as a combined mRS 0–2 group. The same reconstruction algorithm and coding logic were applied at both 90 days and 1 year to ensure consistency across assessments. Further details on the functional domains and operational reconstruction of the mRS are provided in Table S4.

### Statistical analysis

All analyses were performed using IBM SPSS Statistics version 30.0 (IBM) and R version 4.5.3. Baseline characteristics were summarised descriptively using counts (percentages), mean (SD) or median (IQR). Functional trajectories between 90 days and 1 year were defined from within-patient changes in mRS categories (0–2, 3, 4, 5 and 6 [death]) and classified as improved, unchanged, worsened or died. Any transition to a lower mRS category was classified as improvement, whereas transitions to a higher nonfatal category were classified as worsening; death was reported separately. For the primary regression analysis, functional worsening and death were combined into the composite outcome worsening or death. Transitions were summarised using contingency tables and visualised with Sankey diagrams illustrating the direction and magnitude of functional changes over time. Multivariable logistic regression models were used to identify factors associated with worsening or death and improvement between 90 days and 1 year. Separate binary logistic regression models were fitted for worsening or death and improvement. The worsening-or-death model included patients with mRS 0–5 at 90 days, whereas the improvement model was restricted to patients with mRS 3–5 at 90 days. The primary models were adjusted for age, sex, 90-day mRS category, hypertension, atrial fibrillation and diabetes mellitus.^2^ These variables were selected a priori to capture established vascular risk factors and functional status at 90 days, rather than acute baseline variables whose prognostic influence is likely to be strongest during the early post-stroke phase. The NIHSS score was excluded from the primary models because incomplete NIHSS data would have substantially reduced the sample size. Sensitivity analyses additionally adjusted for NIHSS. An additional sensitivity analysis further adjusted the primary models for ICH location as a categorical variable, with deep ICH as the reference category. A further sensitivity analysis restricted to patients alive at 1 year evaluated functional worsening separately; the improvement model was also repeated among 1-year survivors. Complete-case analysis was used for regression models. Patients with missing 1-year follow-up and those lacking 90-day mRS data were analysed separately, with detailed comparisons presented in Table S1. Results are presented as adjusted odds ratios (aORs) with 95% CIs. Two-sided *P*-values < .05 were considered statistically significant.

### Ethics

The study was approved by the Swedish Ethical Review Authority (Dnr 2024-08008-01) and conducted in accordance with the Declaration of Helsinki. In Riksstroke, patients are informed about registration and may opt out; therefore, individual informed consent was not required. Reporting followed the Strengthening the Reporting of Observational Studies in Epidemiology (STROBE) guidelines.^19^

## Results

### Study population and follow-up

Among 13,574 patients with ICH registered between 2017 and 2021, 8583 prestroke-independent patients (prestroke mRS 0–2) remained after exclusion of patients with prestroke dependency or missing prestroke functional status. Of these, 4797 patients were alive and had available mRS data at 90-day follow-up, and 3030 also had complete mRS assessments at 1 year and were therefore included in the primary analysis. A study flowchart illustrating cohort selection is presented in Figure 1.

**Figure 1:**
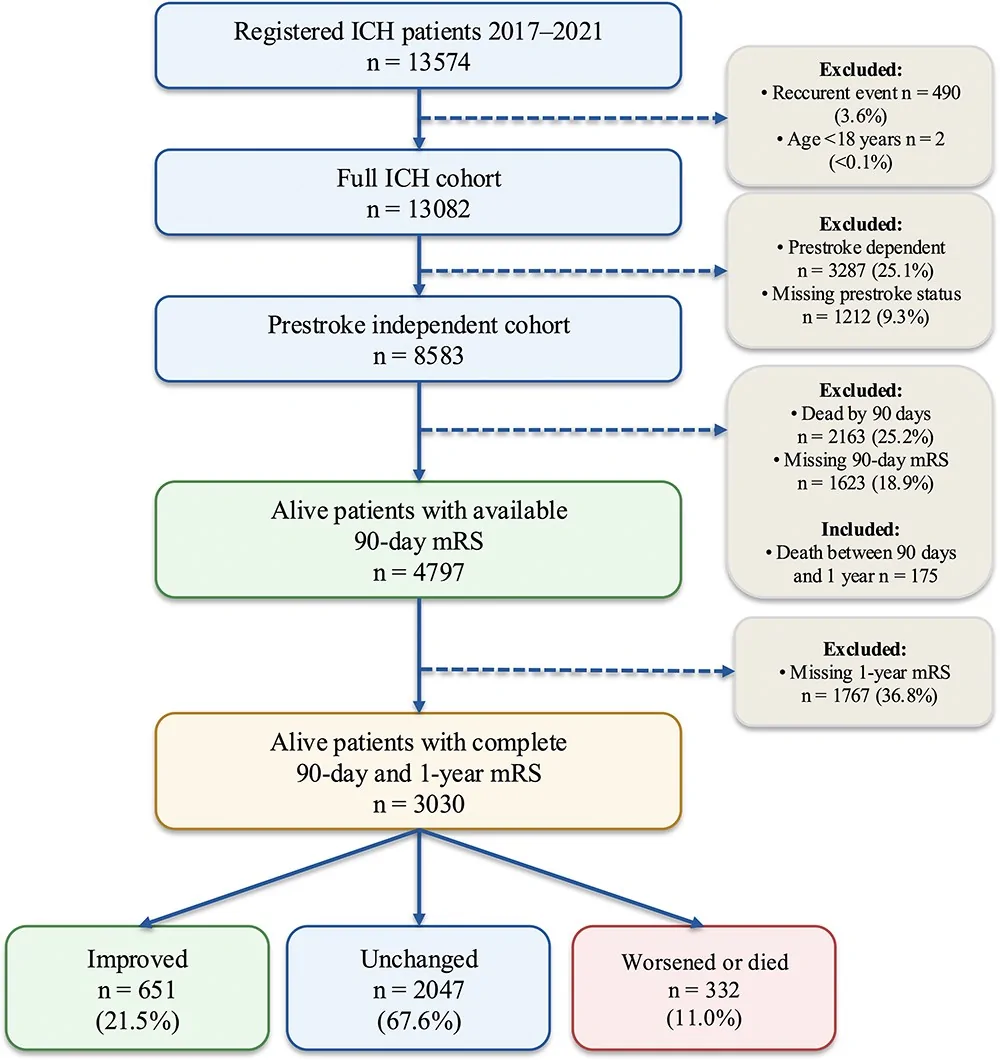
Flowchart of study inclusion and cohort selection. Flowchart illustrating inclusion and exclusion of patients with ICH registered in Riksstroke between 2017 and 2021. The final study cohort consisted of 3030 prestroke-independent patients (prestroke mRS 0–2) who survived to 90 days and had available mRS assessments at both 90 days and 1 year. Functional trajectories between 90 days and 1 year were classified as improved, unchanged, worsened or died according to transitions across grouped mRS categories. Percentages are presented among patients with complete follow-up data at both time points.

### Trajectories of functional outcomes

Functional trajectories between 90 days and 1 year are shown in Figure 2 and Table 1. Overall, 651 patients (21.5%) improved, 2047 (67.6%) remained unchanged, 157 (5.2%) worsened and 175 (5.8%) died. Among the 2855 patients alive at 1 year, 651 (22.8%) improved, 2047 (71.7%) remained unchanged and 157 (5.5%) worsened. Recovery was most frequent among patients with mRS 3 at 90 days, of whom 425/681 (62.4%) were classified as mRS 0–2 at 1 year, whereas 58/681 (8.5%) worsened and 33/681 (4.8%) died. Among patients with mRS 4 at 90 days, 65/434 (15.0%) improved to mRS 0–2 and an additional 59/434 (13.6%) improved to mRS 3, resulting in 28.6% reaching mRS 0–3 at 1 year. Among patients with mRS 5 at 90 days, 23/285 (8.1%) improved to mRS 0–3. Overall, 147/719 (20.4%) of patients with mRS 4–5 at 90 days improved to mRS 0–3 by 1 year, whereas 22/719 (3.1%) worsened and 101/719 (14.0%) died (Table 1). Classification as mRS 0–2 at 90 days was largely maintained, with 1512/1630 (92.8%) remaining in mRS 0–2 at 1 year, whereas 77/1630 (4.7%) worsened and 41/1630 (2.5%) died. Across patients with residual disability at 90 days (mRS 3–5), 498/1400 (35.6%) were classified as mRS 0–2 by 1 year.

**Figure 2:**
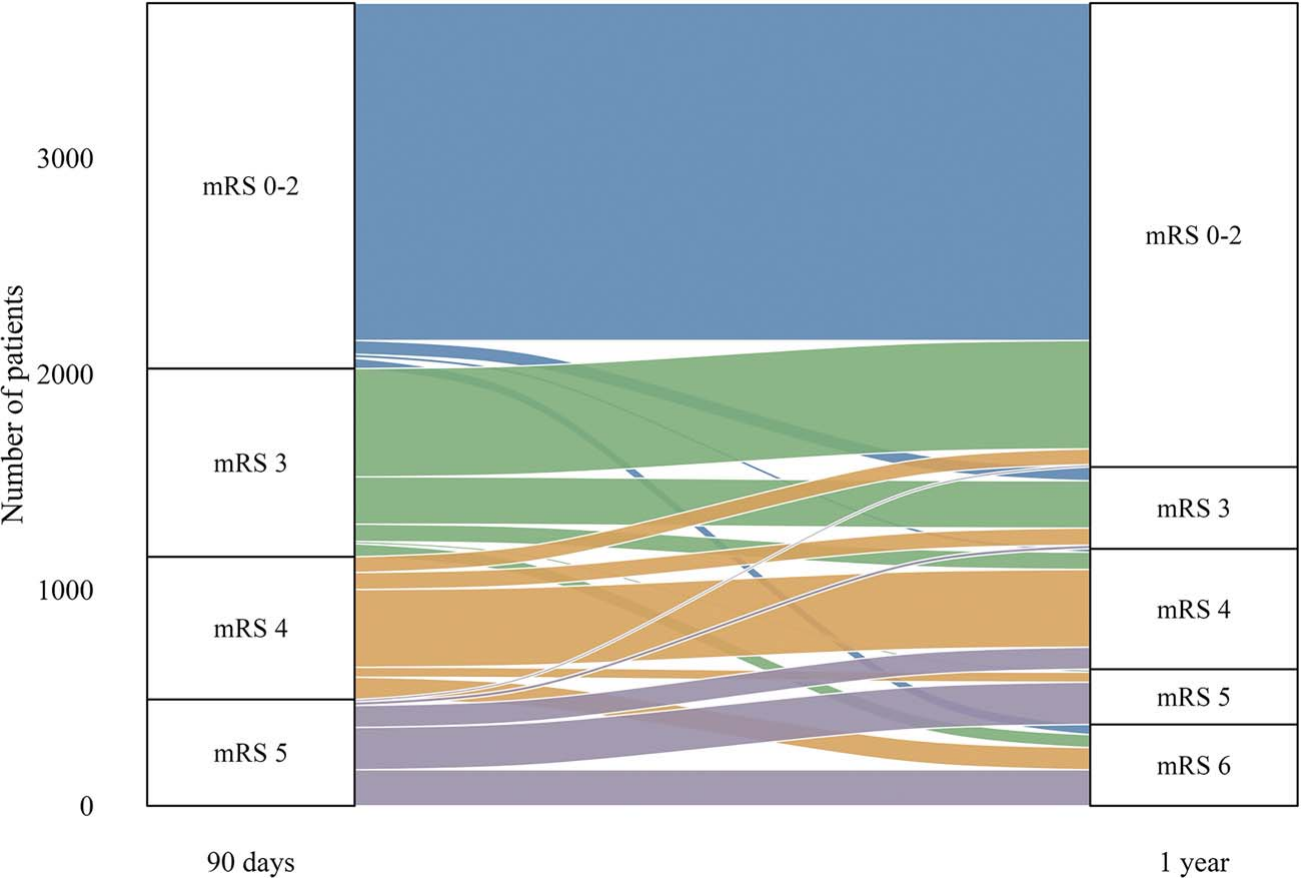
Functional transitions between 90 days and 1 year after intracerebral haemorrhage among prestroke-independent patients. Sankey diagram illustrating transitions in functional status, measured by the mRS, between 90 days and 1 year among 3030 prestroke-independent patients (prestroke mRS ≤ 2) with ICH who survived to 90 days and had available outcome data at both time points. Each flow represents within-patient transitions between mRS categories from 90 days to 1 year.

**Table 1:** Transition in functional outcome between 90 days and 1 year among prestroke-independent patients.

	1-year mRS				
90-day mRS	mRS 0–2	mRS 3	mRS 4	mRS 5	mRS 6
**mRS 0–2**	1512 (92.8%)	59 (3.6%)	15 (0.9%)	3 (0.2%)	41 (2.5%)
**mRS 3**	425 (62.4%)	165 (24.2%)	50 (7.3%)	8 (1.2%)	33 (4.8%)
**mRS 4**	65 (15.0%)	59 (13.6%)	246 (56.7%)	22 (5.1%)	42 (9.7%)
**mRS 5**	8 (2.8%)	15 (5.3%)	79 (27.7%)	124 (43.5%)	59 (20.7%)

Rows represent mRS at 90 days and columns represent mRS at 1 year. Values are presented as row percentages. mRS 0–2 represents the combined lower mRS category used in the reconstruction algorithm; mRS 6 indicates death.

Sensitivity analyses examining alternative assumptions for missing 1-year outcome data demonstrated substantial uncertainty related to incomplete follow-up (Table S2). When all missing outcomes were classified as unchanged, 13.6% improved and 6.9% worsened, whereas classification of all missing outcomes as worsened resulted in 43.8% worsening. Under the most optimistic assumption, in which all missing outcomes were classified as improved, 50.4% were classified as improved.

### Baseline characteristics according to functional trajectory

Baseline characteristics according to functional trajectory are presented in Table 2. Patients with worsening functional status or death between 90 days and 1 year were older than patients who improved or remained unchanged (mean age 75.7 vs 70.4 and 69.0 years, respectively). Prior stroke, atrial fibrillation, hypertension and intraventricular haemorrhage were more common among patients with worsening or death. Patients with worsening or death also had higher NIHSS scores at admission and were less often alert on admission compared with patients who remained unchanged. Neurosurgical treatment was more frequent among patients who improved than among patients with unchanged, worsened or death trajectories. Baseline characteristics according to follow-up status are presented in Table S1. Compared with patients included in the primary analysis, those without complete follow-up more often had prior stroke, severe disability at 90 days, intraventricular haemorrhage, neurosurgical treatment and lower level of consciousness on admission.

**Table 2:** Baseline characteristics by functional trajectory among prestroke-independent 90-day survivors.

Variable	Improved (*n* = 651)	Unchanged (*n* = 2047)	Worsening or death (*n* = 332)	Total (*n* = 3030)
**Demographics**				
**Male sex**	386 (59.3)	1289 (63.0)	185 (55.7)	1860 (61.4)
**Age, mean (SD), years**	70.4 (12.9)	69.0 (12.4)	75.7 (10.3)	70.1 (12.5)
**Living alone before stroke**	242 (37.2)	590 (28.8)	109 (32.8)	941 (31.1)
**Vascular risk factors and medical history**				
**Prior stroke**	80 (12.3)	230 (11.2)	68 (20.5)	378 (12.5)
**Previous TIA or amaurosis fugax**	51 (7.8)	139 (6.8)	31 (9.3)	221 (7.3)
**Atrial fibrillation**	139 (21.4)	382 (18.7)	97 (29.2)	618 (20.6)
**Hypertension**	341 (52.4)	1058 (51.7)	220 (66.3)	1619 (53.5)
**Current smoking**	74 (11.4)	169 (8.3)	30 (9.0)	273 (10.3)
**Diabetes mellitus**	100 (15.4)	277 (13.5)	54 (16.3)	431 (14.2)
**Prior statin use**	183 (28.1)	538 (26.3)	121 (36.4)	842 (27.8)
**Any antiplatelet therapy**	138 (21.2)	355 (17.3)	76 (22.9)	569 (18.8)
**Any OAC therapy**	129 (19.8)	359 (17.5)	93 (28.0)	581 (19.2)
**VKA**	61 (9.4)	168 (8.2)	48 (14.5)	277 (9.1)
**DOAC**	68 (10.4)	191 (9.3)	45 (13.6)	304 (10.0)
**Clinical and stroke characteristics**				
**Alert on admission**	496 (76.2)	1717 (83.9)	260 (78.3)	2473 (82.8)
**Drowsy on admission**	121 (18.6)	251 (12.3)	59 (17.8)	431 (14.4)
**Comatose on admission**	28 (4.3)	49 (2.4)	7 (2.1)	84 (2.8)
**NIHSS score at admission, median (IQR)**	6.0 (2.0–13.0)	3.0 (1.0–8.0)	5.5 (2.0–11.0)	4.0 (1.0–10.0)
**First admitted to stroke unit**	359 (55.2)	1312 (64.1)	227 (68.4)	1898 (63.0)
**Received stroke unit care during acute hospitalisation**	607 (93.2)	1922 (93.9)	306 (92.2)	2835 (93.6)
**Length of hospital stay, median (IQR), days**	23.0 (12.0–45.0)	12.0 (6.0–28.0)	19.0 (9.8–33.0)	15.0 (7.0–32.0)
**Haematoma localisation and acute treatments**				
**Deep ICH**	335 (51.5)	972 (47.5)	152 (45.8)	1459 (49.2)
**Lobar ICH**	99 (15.2)	387 (18.9)	49 (14.8)	535 (18.0)
**Supratentorial unspecified ICH**	91 (14.0)	265 (12.9)	51 (15.4)	407 (13.7)
**Brainstem ICH**	20 (3.1)	77 (3.8)	9 (2.7)	106 (3.6)
**Cerebellar ICH**	61 (9.4)	169 (8.3)	38 (11.4)	268 (9.0)
**Multiple ICH locations**	20 (3.1)	66 (3.2)	17 (5.1)	103 (3.5)
**Other ICH location**	14 (2.2)	68 (3.3)	6 (1.8)	88 (3.0)
**Intraventricular haemorrhage**	181 (27.8)	426 (20.8)	84 (25.3)	691 (23.5)
**Neurosurgical treatment**	81 (12.4)	146 (7.1)	19 (5.7)	246 (8.2)
**Reversal of anticoagulation^a^**	100 (78.7)	270 (76.7)	76 (81.7)	446 (78.0)

Abbreviations: DOAC = direct oral anticoagulant; OAC = oral anticoagulant; VKA = vitamin K antagonist.

Values are presented as no. (%) unless otherwise indicated. Percentages were calculated among patients with available data within each trajectory group; the total column presents prevalence in the overall cohort. Missingness was ≤ 1.5% for all baseline variables except NIHSS at admission (44.3%), current smoking (12.5%), intraventricular haemorrhage (2.9%) and ICH location (2.1%). Reversal of anticoagulation was reported among patients receiving prestroke oral anticoagulation with available reversal data. Continuous variables are presented as mean (SD) or median (IQR).

a Reported among patients receiving prestroke oral anticoagulation.

### Predictors of functional worsening or death and improvement

Multivariable logistic regression analyses are presented in Table 3. Increasing age was associated with higher odds of worsening or death (aOR per year 1.04; 95% CI, 1.03–1.05; *P* < .001) and lower odds of improvement (aOR 0.97; 95% CI, 0.96–0.98; *P* < .001) between 90 days and 1 year. Higher degree of disability at 90 days was also associated with less favourable trajectories. Compared with patients with mRS 0–2 at 90 days, those with mRS 5 had substantially higher odds of worsening or death (aOR 2.56; 95% CI, 1.79–3.63; *P* < .001). In the improvement model, mRS 4 and mRS 5 at 90 days were associated with lower odds of improvement compared with mRS 3 (mRS 4: aOR 0.22; 95% CI, 0.17–0.29; *P* < .001; mRS 5: aOR 0.33; 95% CI, 0.24–0.44; *P* < .001). Male sex was associated with higher odds of improvement (aOR 1.41; 95% CI, 1.12–1.79; *P* = .004), whereas hypertension was associated with lower odds of improvement (aOR 0.73; 95% CI, 0.57–0.94; *P* = .014). Results were broadly unchanged after additional adjustment for NIHSS (Table S3). Additional adjustment for ICH location yielded similar estimates, and ICH location was not significantly associated overall with either worsening or death (*P* = .127) or improvement (*P* = .940) (Table S5). In the sensitivity analysis restricted to patients alive at 1 year, older age remained associated with functional worsening, whereas several associations observed for the composite outcome were attenuated; associations with improvement were broadly similar to the primary analysis (Table S6).

**Table 3:** Multivariable logistic regression analyses for functional trajectories between 90 days and 1 year among prestroke independent patients.

Variable	Worsening or death aOR (95% CI)	*P*-value	Improvement aOR (95% CI)	*P*-value
**Model population**	*n* = 3000		*n* = 1385	
**Age, per year**	1.04 (1.03–1.05)	<.001	0.97 (0.96–0.98)	<.001
**Male sex**	0.85 (0.66–1.08)	.175	1.41 (1.12–1.79)	.004
**90-day mRS category**				
**mRS 3 vs 0–2**	1.53 (1.13–2.07)	.006	…	…
**mRS 4 vs reference**	1.80 (1.28–2.50)^a^	<.001	0.22 (0.17–0.29)^b^	<.001
**mRS 5 vs reference**	2.56 (1.79–3.63)^a^	<.001	0.33 (0.24–0.44)^b^	<.001
**Hypertension**	1.33 (1.03–1.73)	.031	0.73 (0.57–0.94)	.014
**Atrial fibrillation**	1.14 (0.86–1.50)	.346	0.95 (0.71–1.25)	.698
**Diabetes**	1.01 (0.72–1.39)	.948	0.83 (0.60–1.13)	.236

Adjusted odds ratios (aORs) with 95% CIs were estimated using multivariable logistic regression. Models were adjusted for age, sex, 90-day mRS category, hypertension, atrial fibrillation and diabetes. Model populations differed because of missing covariate data.

a Reference category for the worsening-or-death model = mRS 0–2.

b Reference category for the improvement model = mRS 3.

## Discussion

In this large observational study of prestroke-independent 90-day survivors with complete functional follow-up, functional outcomes after ICH remained dynamic beyond 90 days. Approximately one-fifth of patients improved between 90 days and 1 year, whereas 1 in 10 worsened or died. Among patients alive at 1 year, 22.8% improved and 5.5% worsened, indicating that functional change beyond 90 days was not solely explained by mortality. Patients with mRS 3 at 90 days represented a particularly dynamic prognostic subgroup, with more than half achieving mRS 0–2 at 1 year. Classification as mRS 0–2 at 90 days was largely retained, with 1512/1630 (92.8%) remaining in mRS 0–2 at 1 year. In contrast, patients with severe disability at 90 days (mRS 4–5) showed both improvement and worsening over time, although deterioration was predominantly explained by death. Together, these findings suggest that 90-day assessments may incompletely capture longer-term recovery trajectories after ICH.

To our knowledge, this represents the largest study to characterise within-patient functional transitions across the ordinal mRS between 90 days and 1 year after ICH. Our findings support previous reports suggesting that functional recovery after ICH may continue beyond the acute and subacute phases. In a recent multicentre cohort of 458 survivors with supratentorial ICH, 28.6% improved and 17.0% deteriorated between 3 and 12 months, compared with 21.5% and 11.0%, respectively, in the present study.^4^ The somewhat lower recovery rates observed in the current study may partly reflect differences in study populations, as the previous cohort was restricted to supratentorial ICH, whereas the present study included haemorrhages irrespective of location, including infratentorial ICH, which has historically been associated with poorer functional outcomes, particularly cases involving brainstem haemorrhage.^20^^,^^21^ Earlier longitudinal work likewise demonstrated continued recovery during the first year after ICH, with 34% of hospital survivors improving between hospital discharge and 12 months in a prospective cohort of 243 patients.^22^ Although direct comparison is limited by differences in timing of assessment, these findings similarly support the concept that functional recovery after ICH may continue well beyond the acute phase. Importantly, both the present study and a recent study^4^ identified patients with intermediate disability at 3 months as the subgroup with the greatest recovery potential. In the present study, 62.4% of patients with mRS 3 at 90 days achieved mRS 0–2 at 1 year, suggesting substantial recovery potential at this stage after ICH.

Recovery was less frequent and deterioration more common among patients with severe disability at 90 days (mRS 4–5), with 3.1% worsening and 14.0% dying before 1 year. Nevertheless, 20.4% improved to favourable functional outcome (mRS 0–3), suggesting that meaningful recovery remains possible even among patients with substantial residual disability (Table 1). Similar findings have been reported among survivors with severe ICH and intraventricular haemorrhage in pooled analyses of the CLEAR-III and MISTIE-III trials, where more than 40% of patients with mRS 4–5 at 30 days recovered to mRS 0–3 by 1 year, although direct comparison is limited by differences in patient selection and timing of functional assessment (30 vs 90 days).^5^ Continued functional improvement beyond the acute phase is also supported by a post-hoc analysis of the i-DEF trial, in which the proportion of patients achieving favourable functional outcome (mRS 0–2) continued to increase up to 180 days after ICH.^11^ Previous Swedish Riksstroke data from a mixed stroke cohort, predominantly comprising ischaemic stroke, also suggested continued functional change beyond 3 months, with approximately 1 in 6 patients deteriorating between 3 and 12 months after stroke.^10^

Increasing age and greater disability at 90 days were associated with less favourable long-term trajectories after ICH, consistent with previous prognostic studies linking older age to a higher risk of deterioration between 90 days and 1 year after ICH.^10^ Male sex was associated with greater odds of improvement, whereas hypertension was associated with less favourable recovery. The observed association between male sex and improved functional trajectory is consistent with a recent systematic review and meta-analysis reporting better functional outcomes among men after ICH.^23^ Potential explanations for the observed sex difference may include biological differences in inflammatory processes and recovery mechanisms, but also differences in comorbidity, stroke characteristics, rehabilitation, social circumstances and other unmeasured factors. However, these potential explanations cannot be explored with the available data. Although prognostication after ICH remains challenging at the individual level, the present findings may help inform discussions with patients and families regarding longer-term recovery after surviving the acute and subacute phases of ICH. At the group level, these findings may provide cautious reassurance that meaningful recovery can continue after surviving the early phase of ICH, while emphasising that recovery often requires time and remains heterogeneous across patients. The longer hospital stay observed among patients who subsequently improved should be interpreted cautiously, as length of stay may reflect differences in acute care needs, in-hospital recovery, rehabilitation and discharge pathways rather than an independent effect on subsequent recovery. From a clinical perspective, these findings suggest that patients with residual disability may warrant continued follow-up beyond 90 days, including reassessment of rehabilitation needs during the 6- to 12-month period, although our observational data do not establish benefit from renewed rehabilitation during this period.

### Strengths

This study has several strengths. The large cohort and repeated within-patient outcome assessments enabled detailed characterisation of longitudinal functional trajectories after ICH across multiple disability categories rather than dichotomised outcomes. Inclusion of patients treated across all stroke centres in Sweden further enhances the clinical relevance of the findings by reflecting routine clinical practice across a broad healthcare setting. Importantly, the cohort included ICH across all haemorrhage locations and a broad spectrum of stroke severity, rather than selected subgroups. Furthermore, the validity of ICH diagnoses in Riksstroke has recently been radiologically confirmed, supporting the diagnostic accuracy of the study population.^24^ The large sample size further enabled detailed analyses across different 90-day disability strata, allowing characterisation of both recovery and deterioration beyond conventional dichotomised outcomes.

### Limitations

A major limitation relates to incomplete follow-up and the potential for selection bias. First, 1623/8583 (18.9%) prestroke-independent patients had missing 90-day mRS data and were therefore not eligible for longitudinal assessment (Table S1). Compared with patients with available 90-day mRS data, patients without follow-up were younger, more often smokers and less likely to have atrial fibrillation or hypertension, suggesting some differences in case mix. Among 90-day survivors with available mRS data, 1767/4797 (36.8%) lacked 1-year follow-up. Compared with patients included in the primary analysis, patients without 1-year follow-up more often had severe disability at 90 days, prior stroke, intraventricular haemorrhage and neurosurgical treatment (Table S1). The imbalance in baseline characteristics between patients with and without complete follow-up may have led to underestimation of worsening and overestimation of recovery, although baseline characteristics were otherwise broadly comparable between groups. These differences may indicate that loss to follow-up was not entirely random and could therefore have introduced some degree of selection bias, potentially limiting the generalisability of the observed functional trajectories to the broader population of ICH survivors. The wide range of estimates in the extreme-case analyses further illustrates the uncertainty introduced by missing 1-year functional assessments. Second, the primary analyses were restricted to prestroke-independent patients (prestroke mRS 0–2) to more specifically evaluate functional trajectories attributable to the index ICH rather than pre-existing disability. Although this likely improved interpretability of recovery patterns, it reduces generalisability to the broader ICH population. Furthermore, missing prestroke functional information may have been more common among severely affected patients or those who died early, and exclusion of patients without reconstructable prestroke mRS may therefore have introduced selection and survivor bias. Third, the mRS reconstruction algorithm used in Riksstroke^17^ is not currently validated for differentiation between mRS 0, 1 and 2. Consequently, these categories were grouped together as mRS 0–2, preventing assessment of clinically meaningful transitions within the lower disability range. Although the same reconstruction algorithm and coding logic were applied at both follow-up assessments, misclassification between adjacent categories may nevertheless occur and could affect classification of longitudinal transitions. Interobserver variability is a recognised limitation of mRS assessment in general,^25^ and Riksstroke-specific data have also indicated uncertainty in distinguishing adjacent categories, particularly mRS 2 from 3 and mRS 4 from 5.^26^ This is particularly relevant to transitions from mRS 3 to mRS 0–2 and may have influenced the estimated proportion of patients classified as improved. Fourth, NIHSS was incompletely recorded and was therefore not included in the primary multivariable models to avoid substantial loss of sample size and additional selection bias. Nevertheless, sensitivity analyses additionally adjusting for NIHSS showed broadly similar findings (Table S3), supporting the robustness of the main results. Fifth, although the analyses were adjusted for prespecified clinically relevant covariates selected a priori, residual confounding cannot be excluded, and the observational design precludes causal inference. Finally, the study included patients treated between 2017 and 2021, and changes in ICH management since that period may limit the generalisability of these findings to current clinical practice.

### Future directions

Future studies should further determine the optimal timing of functional outcome assessment after ICH beyond the conventional 90-day endpoint. Previous work has suggested that 6-month follow-up may better capture continued recovery after ICH,^11^ although whether the functional changes observed in the present study were already established by 6 months cannot be determined. Our findings therefore suggest that future longitudinal studies should incorporate repeated within-patient assessments across intermediate time points, particularly between 6 months and 1 year, while also extending follow-up beyond 1 year and across different healthcare settings, to better characterise long-term recovery trajectories and help establish a more standardised and optimal timing of outcome assessment for future randomised trials after ICH. Future studies should also seek to better understand the mechanisms underlying functional improvement and deterioration after ICH, including the potential influence of rehabilitation pathways, intensity of post-stroke care and other modifiable factors associated with long-term recovery. From a clinical perspective, the continued recovery observed beyond 90 days supports reassessment of rehabilitation needs during later follow-up, particularly among patients with residual disability. However, whether renewed or intensified rehabilitation between 6 and 12 months improves functional outcome requires evaluation in dedicated interventional studies.

## Conclusion

In this nationwide study of prestroke-independent 90-day ICH survivors with functional assessments available at both 90 days and 1 year, functional status frequently changed between 90 days and 1 year. Patients with moderate disability at 90 days, particularly those with mRS 3, had substantial potential for further recovery, with 62.4% achieving mRS 0–2 by 1 year. In contrast, classification as mRS 0–2 at 90 days was largely maintained, with 92.8% remaining in this category at 1 year. These findings suggest that recovery after ICH continues beyond the conventional 90-day assessment and that longer-term follow-up may provide additional clinically relevant information in observational studies and future ICH trials.

## Supplementary Material

Supplements_aakag129

## Data Availability

Access to aggregated data may be provided upon reasonable request, subject to relevant ethical and regulatory approvals.
