# Supplementary material for: Functional trajectories between 90 days and 1 year after intracerebral haemorrhage: a Swedish Stroke Register (Riksstroke) study
Source: Eur Stroke J. 2026 Oct 28;11(10):aakag129. doi: 10.1093/esj/aakag129 (PMC13715431; doi:10.1093/esj/aakag129)
Supplement: Supplementary file 1 [file supplements_aakag129.docx]

**Supplements:**

**Tables**

**Table S1.** Baseline characteristics according to 90-day and 1-year follow-up status among prestroke-independent patients.

| **Variable** | **90-day mRS follow-up** | | **1-year follow-up among alive patients with available 90-day mRS^a^** | |
| --- | --- | --- | --- | --- |
|  | **Available 90-day mRS (n=6960)** | **Missing 90-day mRS (n=1623)** | **Available 1-year outcome (n=3030)** | **Missing 1-year outcome (n=1767)** |
| Male sex | 4260 (61.2) | 1021 (62.9) | 1860 (61.4) | 1096 (62.0) |
| Age, mean (SD), years | 71.0 (12.8) | 65.0 (15.4) | 70.1 (12.5) | 67.7 (13.8) |
| Living alone before stroke | 2241 (32.3) | 603 (37.6) | 941 (31.1) | 607 (34.6) |
| Prior stroke | 1102 (15.9) | 229 (14.2) | 378 (12.5) | 306 (17.3) |
| Previous TIA or amaurosis fugax | 509 (7.3) | 77 (4.8) | 221 (7.3) | 107 (6.1) |
| Atrial fibrillation | 1596 (23.2) | 285 (17.7) | 618 (20.6) | 323 (18.5) |
| Hypertension | 3844 (55.5) | 809 (50.2) | 1619 (53.5) | 925 (52.4) |
| Current smoking | 639 (11.3) | 211 (15.8) | 273 (10.3) | 189 (12.7) |
| Diabetes mellitus | 1118 (16.1) | 276 (17.1) | 431 (14.2) | 284 (16.1) |
| Prior statin use | 2053 (29.7) | 383 (23.7) | 842 (27.8) | 519 (29.4) |
| Any antiplatelet therapy | 1448 (20.9) | 276 (17.1) | 569 (18.8) | 333 (18.9) |
| Any OAC therapy | 1518 (21.9) | 254 (15.7) | 581 (19.2) | 302 (17.1) |
| **Level of consciousness at admission** | | | | |
| Alert | 4468 (64.9) | 1148 (72.0) | 2473 (82.8) | 1374 (78.8) |
| Drowsy | 1331 (19.3) | 335 (21.0) | 431 (14.4) | 284 (16.3) |
| Comatose | 1081 (15.7) | 111 (7.0) | 84 (2.8) | 85 (4.9) |
| NIHSS score at admission, median (IQR) | 6.0 (2.0–16.0) | 6.0 (2.0–13.0) | 4.0 (1.0–10.0) | 4.0 (2.0–11.0) |
| Admission at Stroke Unit | 3945 (57.0) | 918 (56.7) | 1898 (63.0) | 1084 (61.7) |
| Length of hospital stay, median (IQR), days | 11.0 (5.0–26.0) | 19.0 (8.0–42.0) | 15.0 (7.0–32.0) | 15.0 (7.0–33.0) |
| **ICH location** | | | | |
| Deep | 3474 (51.0) | 754 (47.7) | 1459 (49.2) | 857 (49.6) |
| Lobar | 1115 (16.4) | 282 (17.8) | 535 (18.0) | 328 (19.0) |
| Supratentorial unspecified | 913 (13.4) | 270 (17.1) | 407 (13.7) | 243 (14.1) |
| Brainstem | 277 (4.1) | 50 (3.2) | 106 (3.6) | 55 (3.2) |
| Cerebellum | 567 (8.3) | 130 (8.2) | 268 (9.0) | 151 (8.7) |
| Multiple | 316 (4.6) | 58 (3.7) | 103 (3.5) | 57 (3.3) |
| Other | 155 (2.3) | 37 (2.3) | 88 (3.0) | 36 (2.1) |
| Intraventricular hemorrhage | 2548 (38.0) | 501 (31.9) | 691 (23.5) | 454 (26.8) |
| Neurosurgical treatment | 608 (8.8) | 271 (16.9) | 246 (8.2) | 193 (11.0) |
| **90-day mRS category^b^** | | | | |
| mRS 0-2 | … | … | 1630 (53.8) | 871 (49.3) |
| mRS 3 | … | … | 681 (22.5) | 423 (23.9) |
| mRS 4 | … | … | 434 (14.3) | 269 (15.2) |
| mRS 5 | … | … | 285 (9.4) | 204 (11.5) |

ᵃ Restricted to patients alive with available 90-day mRS data (n=4,797).

ᵇ Only available among patients with recorded 90-day mRS.

ᶜ Values are presented as No. (%) unless otherwise indicated. Continuous variables are presented as mean (SD) or median (IQR). mRS indicates modified Rankin Scale; ICH, intracerebral hemorrhage; NIHSS, National Institutes of Health Stroke Scale; OAC, oral anticoagulant; TIA, transient ischemic attack.

**Table S2.** Sensitivity analyses of functional trajectories under different assumptions for missing 1-year outcome data.

| **Scenario** | **Improved** | **Unchanged** | **Worsening or death** |
| --- | --- | --- | --- |
| **Complete-case primary analysis** | 651/3030 (21.5%) | 2047/3030 (67.6%) | 332/3030 (11.0%) |
| Missing classified as unchanged | 651/4797 (13.6%) | 3814/4797 (79.5%) | 332/4797 (6.9%) |
| Missing classified as worsened | 651/4797 (13.6%) | 2047/4797 (42.7%) | 2099/4797 (43.8%) |
| Missing classified as improved | 2418/4797 (50.4%) | 2047/4797 (42.7%) | 332/4797 (6.9%) |

Values represent the proportion of prestroke-independent patients classified as improved, unchanged, and worsened or death between 90 days and 1 year after ICH under alternative assumptions for missing 1-year mRS data. The primary complete-case analysis included patients with available mRS assessments at both 90 days and 1 year (n=3030). Sensitivity analyses classified all patients with missing 1-year follow-up as unchanged, worsened, or improved, respectively, among 90-day survivors with available 90-day mRS data (n=4797).

**Supplementary Table S3.** Sensitivity analysis additionally adjusted for NIHSS.

| **Variable** | **Worsening or death (n = 1677)** | | **Improvement (n = 767)** | |
| --- | --- | --- | --- | --- |
|  | **aOR (95% CI)** | **p-value** | **aOR (95% CI)** | **p-value** |
| Age, per year | 1.06 (1.04–1.08) | <0.001 | 0.95 (0.93–0.97) | <0.001 |
| Male sex | 0.73 (0.53–1.02) | 0.063 | 1.66 (1.20–2.32) | 0.002 |
| Hypertension | 1.30 (0.91–1.86) | 0.154 | 0.65 (0.46–0.91) | 0.013 |
| Atrial fibrillation | 1.11 (0.76–1.61) | 0.585 | 1.13 (0.78–1.65) | 0.515 |
| Diabetes mellitus | 1.04 (0.66–1.61) | 0.85 | 0.62 (0.40–0.96) | 0.032 |
| NIHSS, per point | 1.01 (0.98–1.04) | 0.54 | 0.97 (0.95–0.99) | 0.016 |
| **90-day mRS (worsening model)** | | |  |  |
| mRS 3 vs 0-2 | 1.41 (0.91–2.16) | 0.121 | … | … |
| mRS 4 vs 0-2 | 1.90 (1.17–3.06) | 0.009 | … | … |
| mRS 5 vs 0-2 | 3.14 (1.79–5.47) | <0.001 | … | … |
| **90-day mRS (improvement model)** | | |  |  |
| mRS 4 vs 3 | … | … | 0.23 (0.15–0.33) | <0.001 |
| mRS 5 vs 3 | … | … | 0.46 (0.29–0.72) | <0.001 |

Adjusted odds ratios (aORs) with 95% confidence intervals were estimated using multivariable logistic regression to identify factors associated with worsening or death and improvement in functional status between 90 days and 1 year after intracerebral hemorrhage. The worsening-or-death model included patients with mRS 0-5 at 90 days, using mRS 0-2 as the reference category, whereas the improvement model included patients with mRS 3-5 at 90 days, using mRS 3 as the reference category. In addition to the main model covariates, this sensitivity analysis further adjusted for stroke severity using NIHSS as a continuous variable. Due to missing NIHSS data, the model populations were reduced to 1,677 patients in the worsening model and 767 patients in the improvement model. Results were broadly consistent with the primary analyses.

**Supplementary Table S4.** Simplified overview of the operational reconstruction of the modified Rankin Scale from Riksstroke functional items

| **Reconstructed mRS** | **Functional information used** |
| --- | --- |
| 0–2 | Independent mobility, toileting and dressing, without substantial dependency on living conditions or support from others |
| 3 | Some dependency in mobility, living conditions, or support from others, while remaining independent in toileting and dressing |
| 4 | Assistance required with mobility, toileting, or dressing |
| 5 | Severe dependency across these functional domains |
| 6 | Death, identified independently through mortality data |

This table provides a simplified overview of how the functional items in Riksstroke are used to reconstruct the mRS categories. The algorithm does not distinguish between mRS 0, 1, and 2, which are therefore represented as a combined mRS 0-2 category. The full algorithm and detailed decision rules have been described previously by Eriksson et al.17, and readers are referred to that publication for further details.

**Supplementary Table S5.** Sensitivity analysis additionally adjusted for ICH location

| **Variable** | **Worsening or death aOR (95% CI)** | **p-value** | **Improvement aOR (95% CI)** | **p-value** |
| --- | --- | --- | --- | --- |
| **Model population** | **n = 2936** |  | **n = 1363** |  |
| Age, per year | 1.04 (1.03–1.06) | <0.001 | 0.97 (0.96–0.98) | <0.001 |
| Male sex | 0.83 (0.65–1.06) | 0.13 | 1.40 (1.10–1.78) | 0.006 |
| **90-day mRS category** | |  |  |  |
| mRS 3 vs 0–2 | 1.44 (1.06–1.97) | 0.02 | … | … |
| mRS 4 vs 0–2 | 1.78 (1.26–2.50) | <0.001 | … | … |
| mRS 5 vs 0–2 | 2.62 (1.82–3.75) | <0.001 | … | … |
| mRS 4 vs 3 | … | … | 0.21 (0.16–0.28) | <0.001 |
| mRS 5 vs 3 | … | … | 0.32 (0.24–0.44) | <0.001 |
| Hypertension | 1.33 (1.02–1.74) | 0.038 | 0.72 (0.56–0.93) | 0.011 |
| Atrial fibrillation | 1.17 (0.88–1.55) | 0.264 | 0.98 (0.74–1.30) | 0.889 |
| Diabetes mellitus | 1.05 (0.75–1.45) | 0.781 | 0.84 (0.61–1.15) | 0.273 |
| **ICH location** | | 0.127^a^ |  | 0.940^a^ |
| Deep | Reference |  | Reference |  |
| Lobar | 0.94 (0.66–1.33) | 0.732 | 1.18 (0.83–1.66) | 0.354 |
| Supratentorial unspecified | 1.28 (0.89–1.80) | 0.172 | 0.99 (0.70–1.41) | 0.96 |
| Brainstem | 1.00 (0.45–1.95) | 0.99 | 1.09 (0.54–2.24) | 0.812 |
| Cerebellar | 1.35 (0.89–2.00) | 0.142 | 0.90 (0.60–1.34) | 0.607 |
| Multiple | 2.03 (1.12–3.51) | 0.014 | 0.84 (0.43–1.61) | 0.597 |
| Other | 0.71 (0.27–1.55) | 0.432 | 0.99 (0.46–2.07) | 0.971 |

Adjusted odds ratios (aORs) with 95% confidence intervals were estimated using multivariable logistic regression. Models were adjusted for age, sex, 90-day mRS category, hypertension, atrial fibrillation, diabetes mellitus, and ICH location. Deep ICH was used as the reference category for ICH location. The worsening-or-death model included patients with mRS 0–5 at 90 days, with mRS 0–2 as the reference category, whereas the improvement model included patients with mRS 3–5 at 90 days, with mRS 3 as the reference category.

^a^ Global likelihood-ratio test for ICH location.

**Supplementary Table S6.** Sensitivity analyses of functional worsening and improvement restricted to patients alive at 1 year

| **Variable** | **Functional worsening, aOR (95% CI), n=2,605** | **P value** | **Improvement, aOR (95% CI), n=1,254** | **P value** |
| --- | --- | --- | --- | --- |
| Age, per year | 1.03 (1.01–1.05) | <0.001 | 0.98 (0.96–0.99) | <0.001 |
| Male sex | 0.76 (0.54–1.06) | 0.107 | 1.39 (1.09–1.78) | 0.009 |
| **90-day mRS category** | |  |  |  |
| mRS 3 vs 0–2 | 1.58 (1.09–2.28) | 0.014 | … | … |
| mRS 4 vs 0–2 | 0.99 (0.59–1.61) | 0.982 | … | … |
| mRS 4 vs 3 | … | … | 0.22 (0.17–0.29) | <0.001 |
| mRS 5 vs 3 | … | … | 0.42 (0.30–0.57) | <0.001 |
| Hypertension | 1.16 (0.81–1.67) | 0.409 | 0.75 (0.58–0.97) | 0.026 |
| Atrial fibrillation | 1.00 (0.66–1.49) | 0.991 | 1.03 (0.76–1.38) | 0.858 |
| Diabetes mellitus | 0.94 (0.58–1.48) | 0.811 | 0.90 (0.65–1.25) | 0.529 |

Analyses were restricted to patients alive at 1 year. Models were adjusted for age, sex, 90-day mRS category, hypertension, atrial fibrillation, and diabetes mellitus. The functional worsening model included patients with mRS 0–4 at 90 days; patients with mRS 5 were excluded because further nonfatal worsening on the grouped mRS was not possible. The improvement model was restricted to patients with mRS 3–5 at 90 days. Complete-case analysis was used.
